# Supplementary material for: Gliotic Response and Reprogramming Potential of Human Müller Cell Line MIO-M1 Exposed to High Glucose and Glucose Fluctuations
Source: Int J Mol Sci. 2024 Nov 29;25(23):12877. doi: 10.3390/ijms252312877 (PMC11641291; doi:10.3390/ijms252312877)
Supplement: Supplementary file 1 [file ijms-25-12877-s001.zip › ijms-3335673-supplementary.pdf]

Supplementary Materials

Gliotic Response and Reprogramming Potential of Human Müller Cell Line MIO-M1 Exposed to High Glucose and Glucose Fluctuations †

Benedetta Russo [1,‡], Giorgia D'Addato [2,‡], Giulia Salvatore [3], Marika Menduni [1], Simona Frontoni [4], Luigi Carbone [5], Antonella Camaioni [3], Francesca Gioia Klinger [2], Massimo De Felici [3], Fabiana Picconi [1,*] and Gina La Sala [3,6]

[1] Unit of Endocrinology and Diabetology, Isola Tiberina-Gemelli Isola Hospital, 00186 Rome, Italy; benedetta_russo6@msn.com (B.R.); marika.menduni@gmail.com (M.M.)

[2] Section of Histology and Embryology, Saint Camillus International University of Health Sciences, 00131 Rome, Italy; giorgia.daddato@alumni.uniroma2.eu (G.D.); klinger@uniroma2.it (F.G.K.)

[3] Department of Biomedicine and Prevention, University of Rome Tor Vergata, 00133 Rome, Italy; giulias@mclink.it (G.S.); camaioni@uniroma2.it (A.C.); defelici@uniroma2.it (M.D.F.); gina.lasala@cnr.it or gina.la.sala@uniroma2.it (G.L.S.)

[4] Department of Systems Medicine, University of Rome Tor Vergata, 00133 Rome, Italy

[5] Unit of Emergency Room, Emergency Medicine and Internal Medicine, Isola Tiberina-Gemelli Isola Hospital, 00186 Rome, Italy; luigi.carbone@fbf-isola.it

[6] CNR Institute of Biochemistry and Cell Biology, 00015 Rome, Italy

* Correspondence: fabipicco@gmail.com

† We dedicate this article to Simona Frontoni, a great woman, teacher, and researcher who allowed us to create and carry out this research project with her enthusiasm and passion for research in the field of diabetes and diabetic neuropathy.

‡ These two authors contributed equally to this work.

Supplementary Materials

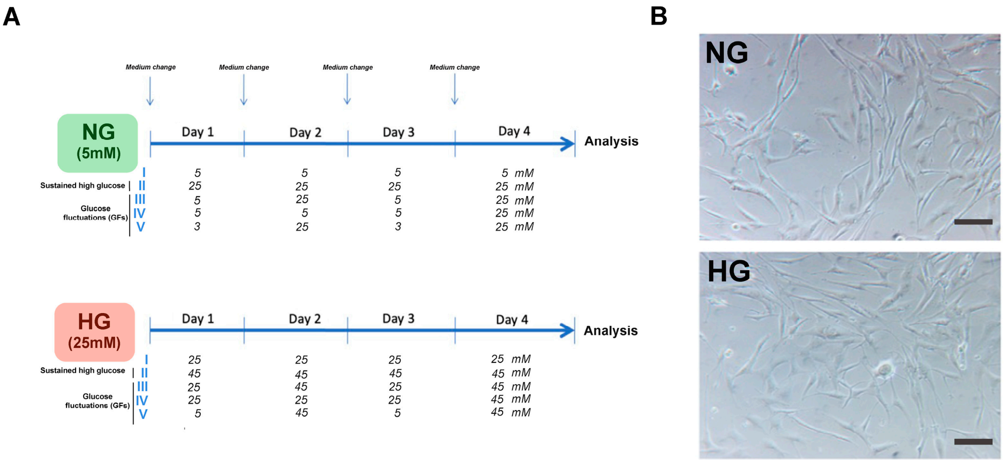

Figure S1. Experimental Design. (**A**) NG and HG MIO-M1 cells were maintained in basal glucose medium (treatment I) or exposed to a sustained high glucose (treatment II) and glucose fluctuations (GFs) (treatments III-V) as illustrated in the panel. (**B**) Representative Bright-Fields images of MIO-M1 cells grown in NG and HG basal conditions. Scale bars: 100 µm.
